# Neurophysiological markers of early cognitive decline in older adults: a mini-review of electroencephalography studies for precursors of dementia

**DOI:** 10.3389/fnagi.2024.1486481

**Published:** 2024-10-18

**Authors:** Mutsuhide Tanaka, Emi Yamada, Futoshi Mori

**Affiliations:** ^1^Department of Health and Welfare Occupational Therapy Course, Faculty of Health and Welfare, Prefectural University of Hiroshima, Hiroshima, Japan; ^2^Department of Linguistics, Faculty of Humanities, Kyushu University, Fukuoka, Japan

**Keywords:** electroencephalogram (EEG), event-related potentials (ERPs), dementia prevention, neurophysiological biomarker, mild cognitive impairment, subjective cognitive decline, subjective memory complaint, cognitive frailty

## Abstract

The early detection of cognitive decline in older adults is crucial for preventing dementia. This mini-review focuses on electroencephalography (EEG) markers of early dementia-related precursors, including subjective cognitive decline, subjective memory complaints, and cognitive frailty. We present recent findings from EEG analyses identifying high dementia risk in older adults, with an emphasis on conditions that precede mild cognitive impairment. We also cover event-related potentials, quantitative EEG markers, microstate analysis, and functional connectivity approaches. Moreover, we discuss the potential of these neurophysiological markers for the early detection of cognitive decline as well as their correlations with related biomarkers. The integration of EEG data with advanced artificial intelligence technologies also shows promise for predicting the trajectory of cognitive decline in neurodegenerative disorders. Although challenges remain in its standardization and clinical application, EEG-based approaches offer non-invasive, cost-effective methods for identifying individuals at risk of dementia, which may enable earlier interventions and personalized treatment strategies.

## 1 Introduction

Rapid aging of the global population has intensified the need to extend healthy life expectancy, and dementia poses an important challenge to this goal. Alzheimer’s disease (AD) and other types of dementia are characterized by cognitive decline that is distinct from that of normal aging, necessitating a deeper understanding of the underlying mechanisms. Recent research has revealed that AD-associated pathophysiological changes can begin more than a decade before the onset of clinical symptoms (Ritchie et al., 2016; Moffat et al., 2022). Although postmortem examination remains the definitive method for diagnosing dementia, important advancements in *in vivo* assessment techniques have emerged, including cerebrospinal fluid biomarkers, positron emission tomography, and magnetic resonance imaging (MRI) (Clark et al., 2018; Hojjati et al., 2018; Liu et al., 2024). However, these methods present various challenges, such as high cost, invasiveness, and limited clinical accessibility.

Electroencephalography (EEG) offers a non-invasive, cost-effective approach for detecting neurological markers of cognitive decline. Recent reviews have focused on EEG characteristics in AD and mild cognitive impairment (MCI) (Al-Qazzaz et al., 2014; Sanchez-Reyes et al., 2021; Torres-Simon et al., 2022; Wijaya et al., 2023). EEG activity correlates with cognitive decline assessed by the Mini-Mental State Examination (MMSE), and combining these measures improves dementia prediction accuracy (Doan et al., 2021). EEG may detect subtle early functional changes. However, research on EEG markers of early precursors, such as subjective cognitive decline (SCD), subjective memory complaints (SMC), and cognitive frailty (CF), remains scarce.

This mini-review summarizes recent EEG findings used to identify a high risk of dementia in older adults, emphasizing conditions such as SCD, SMC, and CF. We explore EEG-based approaches, including event-related potentials (ERPs), quantitative EEG (qEEG) markers, microstate analysis, and functional connectivity measures. Additionally, we discuss the integration of EEG with artificial intelligence technologies for early diagnosis and prediction of dementia progression.

By focusing on pre-MCI states, we aim to increase knowledge of the early detection of cognitive decline, thus enabling earlier interventions and more effective prevention strategies. Additionally, we highlight the challenges and future directions in this field, emphasizing the need for standardized approaches and larger-scale studies to validate the clinical utility of EEG-based markers in dementia risk assessments.

## 2 Early cognitive decline: from normal aging to pre-MCI states

The risk of dementia in older adults is influenced by 12 modifiable risk factors (Livingston et al., 2020). Previous studies have pointed out the association between preclinical stages of AD (i.e., SMC and SCD) and these lifestyle risk factors, such as low education and hypertension (Chen et al., 2014), and depression and cigarette smoking (Ahn et al., 2021). The importance of treating these factors before cognitive decline onset or at the subjective complaint stage is increasingly emphasized (Van Der Flier et al., 2023).

The spectrum of cognitive decline ranges from normal aging to dementia, encompassing crucial intermediate stages for early detection and intervention. MCI is a high-risk state for progression to dementia, particularly AD (Arnáiz and Almkvist, 2003), and is characterized by clinical symptoms, minimal assistance needs with daily activities, and potentially reversible cognitive decline (García et al., 2021).

Recent studies have focused on earlier stages of cognitive decline. In SCD and SMC, individuals experience self-perceived cognitive decline but perform within the normal range on objective tests, and exhibit an increased risk of progressing to MCI and dementia (Kryscio et al., 2014; Bessi et al., 2018). CF represents coexisting physical frailty and MCI, and encompasses mild cognitive decline even without a diagnosed neurological disorder (Kelaiditi et al., 2013; Shimada et al., 2018; Facal et al., 2021). Kocagoncu et al. (2022) defined CF as mild cognitive decline without subjective awareness, indicating that the concept of CF is not fully established. CF is linked to increased risks of dementia, care needs, hospitalization, disability, and mortality compared with healthy aging (Lee et al., 2018; Panza et al., 2018).

Distinguishing these early stages from normal aging is challenging because differences can be subtle and not always apparent using standard cognitive assessments. EEG primarily reflects postsynaptic potentials, offering promising avenues for identifying early markers of cognitive decline. EEG may detect subtle changes in postsynaptic fields that potentially underlie cognitive dysfunction in AD and MCI (Arendt, 2009; Targa Dias Anastacio et al., 2022).

## 3 Contemporary ERP methodologies and their application

While EEG may reflect postsynaptic potentials and neuronal population activity, ERPs are derived from averaging electrical responses to specific stimuli or tasks, enabling identification of components related to perception and cognition. Goodin et al. (1978) first identified the P300 component as a biomarker for dementia, characterized by a positive waveform occurring 200–300 ms after an oddball task event. AD typically results in attenuated P300 amplitude and increased latency compared with normal aging (Pedroso et al., 2012; Hedges et al., 2016; Fruehwirt et al., 2019). The P300 is also sensitive to MCI; reduced P300 amplitude indicates cognitive deterioration in at-risk older adults (Newsome et al., 2013), and its latency may predict MCI progression to AD (Jiang et al., 2015).

Table 1 summarizes recent ERP studies on early cognitive decline in older adults. Evidence regarding the P300 in SCD and SMC is limited but promising. People with SMC progressing to AD show a prolonged P300 latency before AD onset (Gironell et al., 2005) and in response to stimulus–response incongruence (Cespón et al., 2018). Ulbl and Rakusa (2023) reviewed studies that demonstrated decreased N170 and P300 amplitudes in SCD, although the results across ERP components were inconsistent. The P3b is a later component of the P300, and has exhibited decreased amplitude in cognitively low-performing older adults, suggesting age-independent episodic memory decline (Porcaro et al., 2019). Additionally, P300 peak amplitude correlates with bilateral hippocampal volume in healthy older adults (Devos et al., 2021).

**Table 1:** Summary of ERP studies of early cognitive decline in older adults.

Authors (Year)	Participants	ERP task	ERP component	Amplitude effects	Latency effects	Other effects
Gironell et al. (2005)	SMC (*n* = 116)	Oddball	P300	–	AD > NC, MCI, DOT	Baseline P300 latency predicted AD diagnosis
Cespón et al. (2018)	Low SMC (*n* = 18), High SMC (*n* = 16)	Simon task	P300, MFN	High SMC: larger MFN for incompatible trials	P300: longer for incompatible position	High SMC: interference from arrow direction at slow RTs
Ulbl and Rakusa (2023)	SCD, MCI, AD, NC (Review of 30 studies)	Various	P300, N170	SCD: reduced P300/N170 amplitudes in some studies	SCD: increased P300/N170 latencies in some studies	EEG: SCD showed slowing of rhythms and connectivity changes
Porcaro et al. (2019)	Young (*n* = 15), HP Old (*n* = 17), LP Old (*n* = 14)	Visual three-stimulus oddball	P3a, P3b	P3b: Young > HP > LP P3a: Young > HP, LP	P3a, P3b: Young< HP, LP	FSS improved detection of group differences; P3b amplitude distinguished HP from LP
Cheng et al. (2021)	SCD (*n* = 26), NC (*n* = 29)	Not specified	MMNm	SCD < HC in left IPL and right IFG	–	MMNm amplitudes in right IFG correlated with memory performance in SCD; No GM volume differences between groups
Pei et al. (2020)	SCD (*n* = 17)	Auditory oddball	MMN	Increased at Pz after training	–	Improved WM performance, especially in auditory tone 3-back task
Tarawneh et al. (2023)	SMC (*n* = 43), non-SMC (*n* = 19)	Auditory oddball	P50, N100, P200, N200, P300	–	P50: Aβ+ > Aβ-; P50 latency weakly correlated with MAC-Q scores	P50 latency may identify individuals at higher risk of cognitive decline
Garrido-Chaves et al. (2021)	Young SMC (*n* = 28), Young noSMC (*n* = 37), Older SMC (*n* = 32), Older noSMC (*n* = 39)	Iowa Gambling Task	FRN, P3	FRN: Losses > Wins; Older > Young; P3: Young > Older	FRN, P3: Older > Young; FRN: Older SMC > Older noSMC for losses in first block	Older SMC showed worse behavioral performance in ambiguity phase
Kocagoncu et al. (2022)	CF (*n* = 26), NC (*n* = 38), MCI (*n* = 15), AD (*n* = 11)	Cross-modal oddball	MMN	CF, NC > MCI, AD for novel and associative deviants	–	CF showed similar neurophysiological profile to NC, despite poor cognitive performance

Aβ, amyloid-β; AD, Alzheimer’s disease; AERP, auditory event-related potential; CF, cognitive frailty; DOT, dementia of other type; ERP, event-related potentials; FRN, feedback-related negativity; FSS, functional source separation; GM, gray matter; HC, healthy control; HP, high performing; IFG, inferior frontal gyrus; IPL, inferior parietal lobule; LP, low performing; MAC-Q, Memory Assessment Clinics Questionnaire; MCI, mild cognitive impairment; MFN, medial frontal negativity; MMN, mismatch negativity; MMNm, magnetic mismatch negativity; NC, normal control; RT, reaction time; SCD, subjective cognitive decline; SMC, subjective memory complaints; WM, working memory.

Mismatch negativity (MMN) reflects the automatic detection of sensory input changes. Attenuated MMN is associated with memory and psychosocial deficits (Mowszowski et al., 2012) and is decreased in AD and MCI compared with normal aging (Kazmerski et al., 1997; Papadaniil et al., 2016). The neural sources of MMN show a characteristic migration pattern with AD progression (Papadaniil et al., 2016; Tsolaki et al., 2017). Ruzzoli et al. (2016) reported distinctive patterns of auditory MMN distribution in normal aging, MCI, and AD. In SCD, magnetoencephalography (MEG)-measured MMN revealed that attenuated responses were correlated with memory function (Cheng et al., 2021). Additionally, MMN-based neurofeedback is reportedly effective for working memory training in SCD (Pei et al., 2020).

The N200 component has shown utility for differentiating MCI from AD (Papaliagkas et al., 2009b; Morrison et al., 2018) and predicting progression risk to MCI/AD in healthy older adults (Papaliagkas et al., 2009a; Howe, 2014). Although similar effects in N400 and P600 have been reported (Grieder et al., 2013; Chou et al., 2023), their usefulness remains unclear in the context of SCD, SMC, and CF.

Research on other ERP components has been limited. Tarawneh et al. (2023) reported prolonged P50 latency in amyloid-*β*-positive participants compared with healthy controls. Changes in ERPs during cognitive tasks have been reported in SMC, including prolonged feedback-related negativity latencies (Garrido-Chaves et al., 2021). Kocagoncu et al. (2022) proposed that CF is part of the normal neurocognitive spectrum, as its MMN responses resemble those of normal aging.

Cognitive function in the normal range, possibly resulting from compensatory neural mechanisms (Sala-Llonch et al., 2015; Wei et al., 2022), may contribute to low sensitivity to ERP components in SMC and SCD. Thus, EEG may offer more sensitive and valuable information regarding early cognitive decline than ERPs in SCD, SMC, and CF.

## 4 Exploring the frontiers of EEG research: advanced approaches to elucidating early cognitive decline as a risk for dementia

### 4.1 Quantitative EEG markers during precursor symptoms of AD

qEEG analyzes digital EEG signals using mathematical algorithms (Nuwer, 1997), providing insights into potential early neurobiomarkers of pathological cognitive aging (Keller et al., 2023). Unlike ERPs focusing on time-locked responses, qEEG examines ongoing EEG activity, offering a broader view of brain function. qEEG includes linear techniques, including power spectral analysis, and nonlinear methods, including entropy measurements and fractal dimension analysis (Al-Qazzaz et al., 2014). In AD, EEG typically shows reduced alpha and beta band activity (Wada et al., 1998; Knott et al., 2000), distinct from normal aging (Babiloni et al., 2021).

Recent research focuses on differences between normal aging and prodromal AD pathophysiology, including MCI, SCD, and SMC. A key finding in MCI and AD is “EEG slowing,” in which increased occipital low-frequency power and decreased frontal high-frequency power are correlated with cognitive performance (Farina et al., 2020; Medici et al., 2023). The theta/alpha ratio indicates cognitive decline, showing differences between AD, MCI, and healthy older adults (Meghdadi et al., 2021), with an increased ratio in MCI associated with higher dementia risk (Hamilton et al., 2021).

EEG slowing parameters show promise for detecting early cognitive decline in SCD and SMC (Table 2). Previous studies have reported decreased frontal EEG slowing parameters with declining cognitive scores in healthy older adults (Choi et al., 2019), increased theta power and reduced alpha reactivity in SMC (Perez et al., 2022), and altered oscillatory activity in SCD (Shim et al., 2022).

**Table 2:** Summary of EEG studies of early cognitive decline in older adults.

Authors (Year)	Participants	EEG task	Frequency bands	Power/amplitude effects	Functional connectivity	Other features	Main findings
Choi et al. (2019)	496 elderly (165 Male, 331 Female), age ≥ 50 years	Resting-state, eye closed	*α*, *θ*	MF, PF, TAR ↓ with lower MMSE	–	EEG from Peak Frequency (Fp1, Fp2)	(1) MDF, PF, TAR: correlated with MMSE (2) EEG slowing significantly between MMSE T2 vs. T1
Shim et al. (2022)	SMC (*n* = 95): 26 A+, 69 A−, age ≥ 65 years	Resting-state, eye closed	*δ*, θ, *α*1, α2, β1, β2, β3, *γ*	A+: (1) ↑ relative *δ* in F, P, O (2) ↓ relative α1 in F, C, O	↑ connections bilateral PCu in δ ↓ connections bilateral entorhinal areas in α1	19 scalp electrodes; sLORETA; DMN analysis	(1) A+: ↑δ, ↓α1 (2) ↓α1 in bilateral fusiform & inferior temporal area, ↑δ in posterior regions
Babiloni et al. (2020)	SMC (*n* = 172): 118 A−, 54 A+, age ≥ 70 years	Resting-state, eye closed	δ, θ, α1, α2, α3, β1, β2, γ	A+ high education: ↓ O α2, ↑ T α3 A− high education: ↑ P, O, T α2 & α3	–	19 scalp electrodes; IAF-based analysis	(1) A− high education: ↑ posterior α (2) A+ high education: ↑ T α3, ↓ O α2
Lopez et al. (2024)	SMC (*n* = 161): 105 A−, 56 A+, age ≥ 70 years	Resting-state, eye closed	δ, θ, α1, α2, α3, β1, β2, γ	A− high education: ↑ P, O, T α2, ↑ O α3 A+ high education: ↓ F, O α2 & α3	+ associations Thal-VN connections & posterior α3 in A− high education	68 scalp electrodes; rs-fMRI; amyPET	(1) A− high education: ↑ posterior α (2) A+ high education: ↓ posterior α
Engedal et al. (2020)	SMC (*n* = 45), MCI (*n* = 88), NC (*n* = 67), age ≥ 50 years	Resting-state, eye closed	–	–	–	qEEG using SPR method; DI (0–100)	DI predicted conversion to dementia with moderate accuracy (AUC = 0.78)
Spinelli et al. (2022)	SMC (*n* = 318): 230 A−, 88 A+, age 70–85 years	Resting-state, eye closed	δ, θ, α1, α2, β1, β2, γ	Baseline: A+ ↑ MF θ 24-month follow-up: A+ ↑ PC θ, ↓ O α1	–	256 electrodes; source-level analysis; longitudinal	(1) A+: ↑ MF θ at baseline, ↑ PC θ at follow-up (2) Suggests DMN hypoactivation in A+
Lassi et al. (2023)	SCD (*n* = 57), MCI (*n* = 46), NC (*n* = 19)	Resting-state	δ, θ, α, β	↑ δ power in MCI vs. NC in left central ROI	SWI in δ band: SCD > MCI	Microstates analysis, LZ complexity, Hurst exponent	(1) Microstate C: ↓ duration and coverage in MCI vs. NC and SCD (2) ↓ LZ complexity in MCI vs. SCD (3) Hurst exponent: NC > SCD > MCI (4) Microstate C topography different in AD-like CSF profile
Shi et al. (2022)	AD (*n* = 13), MCI (*n* = 19)	Resting-state	2–20 Hz (secondary filter)	–	–	Microstate parameters (GEV, TPs, TTPs)	(1) AD showed longer microstate durations and fewer occurrences than MCI. (2) TPC → A-D → A correlated with MMSE scores (negatively in AD, positively in MCI). (3) Using TTPs and Partial Accumulation strategy, LDA classifier achieved 93.8% accuracy in distinguishing AD from MCI
López-Sanz et al. (2017)	SCD (*n* = 41), MCI (*n* = 51), NC (*n* = 39)	Resting-state	α (6.9–11.4 Hz)	–	Whole-brain FC analysis; DMN and DAN analysis	PLV, SWI	(1) SCD and MCI showed similar FC alterations: ↑ FC in anterior network, ↓ FC in posterior network. (2) MCI had more pronounced posterior FC decrease vs. SCD. (3) ↓ FC in DAN and posterior DMN for both SCD and MCI vs. HC. (4) FC changes correlated with cognitive scores and hippocampal volume.
Cheng et al. (2020)	SCD (*n* = 27), NC (*n* = 26)	Resting-state	δ, θ, α, β, γ1, γ2	–	↑ FC in DMN for SCD vs. NC in δ and γ bands	AEC, Node strength	(1) ↑δ band FC in SCD between LTC-PCC and PCu-PCC. (2) ↑γ band FC in SCD between LTC-PCC and PCu-PCC. (3) PCC node strength in δ and γ bands showed good discrimination ability for SCD vs. NC (AUC > 0.75). (4) PCC γ1 node strength correlated with cognitive complaints in SCD.
Hou et al. (2018)	Young (*n* = 15, 19–29 years), Senior (*n* = 10, 58–70 tears)	Resting-state, 0-back, 2-back	θ, α, β, γ	–	PLI	Clustering coefficient, Characteristic path length, Small-world coefficient	(1) Age-related alterations more prominent in 2-back task, especially in θ band. (2) ↑θ band FC and nodal clustering coefficient in seniors during 2-back. (3) ↓α band small-world coefficient in seniors during both *n*-back tasks. (4) Young adults showed ↑β band clustering coefficient during 2-back vs. rest; absent in seniors. (5) θ and γ band metrics correlated with working memory performance.
Kim et al. (2021)	SCD (*n* = 180), MCI (*n* = 63)	Resting-state, eyes-closed	δ, θ, α1, α2, β1, β2, β3, γ	Various power changes reported	–	Relative power, Genetic algorithm for feature selection, Multi-model ensemble	(1) SCD amyloid classification: 85.7% sensitivity, 89.3% specificity, 88.6% accuracy. (2) MCI amyloid classification: 83.3% sensitivity, 85.7% specificity, 84.6% accuracy. (3) Genetic algorithm identified optimal EEG features for classification. (4) Multi-model ensemble approach improved classification performance.

A−, amyloid PET-negative; A+, amyloid PET-positive; AD, Alzheimer’s disease; AEC, amplitude envelope correlation; amyPET, amyloid positron emission tomography; AUC, area under the curve; C, central; CSF, cerebrospinal fluid; DAN, dorsal attention network; DI, dementia index; DMN, default mode network; EEG, electroencephalography; F, frontal; FC, functional connectivity; GEV, global explained variance; IAF, individual alpha frequency; LDA, linear discriminant analysis; LTC, lateral temporal cortex; LZ, Lempel–Ziv; MCI, mild cognitive impairment; MDF, median frequency; MF, midfrontal; MMSE, Mini-Mental State Examination; NC, normal control; O, occipital; P, parietal; PC, posterior cingulate; PCC, posterior cingulate cortex; PCu, precuneus; PF, peak frequency; PLI, phase lag index; PLV, phase locking value; qEEG, quantitative EEG; ROI, region of interest; rs-fMRI, resting-state functional magnetic resonance imaging; SCD, subjective cognitive decline; sLORETA, standardized low-resolution brain electromagnetic tomography; SMC, subjective memory complaints; SPR, statistical pattern recognition; SWI, small world index; T, temporal; TAR, theta-alpha ratio; Thal-VN, thalamus-visual network; TPs, transition probabilities; TTPs, time-factor transition probabilities.

Higher education levels are correlated with higher posterior alpha rhythm amplitudes in SMC (Babiloni et al., 2020) and enhanced neural coupling between posterior alpha rhythm and thalamus-visual networks (Lopez et al., 2024), suggesting a protective role of cognitive reserve.

Several studies have demonstrated qEEG’s potential for predicting progression from preclinical to AD. Engedal et al. (2020) reported moderate accuracy in predicting transition to dementia in SMC and MCI. Associations between qEEG parameters and pathological protein biomarkers suggest that resting-state EEG changes might reflect increased brain amyloid burden in AD progression (Spinelli et al., 2022; Ulbl and Rakusa, 2023).

Nonlinear methods have shown promising results in distinguishing AD patients from healthy older individuals (Abásolo et al., 2006; Pineda et al., 2020), potentially capturing complex brain dynamics not evident in linear analyses. However, studies employing nonlinear techniques for SMC and SCD have been limited, mainly using MEG (e.g., Shumbayawonda et al., 2020). The application of this approach to preclinical dementia stages faces challenges, including high computational costs and complex data interpretation (Vicchietti et al., 2023).

Although EEG biomarkers exist for SCD and SMC, research examining CF remains limited. Some studies have suggested that CF exhibits brain activity patterns related to physical conditions (Suárez-Méndez et al., 2021) linked to cognitive function (Liu et al., 2024). CF characteristics may be discerned through changes in cognitive function-related neural oscillations, microstate analysis, functional connectivity, and phase coherence analysis.

### 4.2 Integrating microstate and connectivity analyses for the early detection of cognitive decline

Although qEEG provides insights into frequency characteristics of resting-state brain activity, advanced techniques like microstate analysis, functional connectivity assessment, and graph theory approaches offer a deeper understanding of brain network dynamics in cognitive decline. These methods show promise for differentiating normal aging from pathological changes, including AD and prodromal AD symptoms.

Microstate analysis captures functional network dynamics with millisecond-level resolution, revealing distinct characteristics between AD and MCI. EEG microstates, brief periods of quasi-stable scalp electrical patterns typically classified into four topographies (A–D), reflect momentary global brain states and the basic units of cognitive processing (Michel and Koenig, 2018). Significant differences in microstate topographies—particularly A, C, and D—between healthy controls and AD/MCI (Britz et al., 2010; Smailovic et al., 2019; Lian et al., 2021) may reflect dysfunction in key brain networks (e.g., default mode network or frontoparietal network) associated with AD pathology.

Changes in microstate dynamics have been observed in MCI and AD. Musaeus et al. (2019, 2020) reported higher transition probabilities from microstates C and D to A, and increased occurrence frequencies and coverage of microstate A, in AD and MCI compared with healthy controls. Notably, Lassi et al. (2023) found reduced complexity of microstate transitions in MCI and SCD, indicating simpler brain network dynamics even at the SCD stage. Shi et al. (2022) reported that specific microstate transition probabilities (C → A − D → A) correlate with MMSE scores, suggesting applications for identifying potential cognitive impairment and brain activity patterns in the pre-dementia stage.

Functional connectivity analysis provides insights into SCD and MCI pathophysiology without apparent structural changes. López-Sanz et al. (2017) identified anterior network hyper-synchronization and decreased posterior network connectivity in SCD and MCI during the resting state. Cheng et al. (2020) reported increased functional connectivity within the default mode network in the delta and gamma frequency bands in SCD using MEG, potentially representing compensatory mechanisms.

Graph theory approaches have further elucidated changes in brain network organization across the cognitive decline spectrum (Rubinov and Sporns, 2010). Vecchio et al. (2014) applied graph theory to EEG analysis, revealing differences in brain networks between healthy elderly and AD patients. EEG of normal subjects showed high interaction between channels, while AD patients exhibited more random brain network structures, particularly in the alpha band. These changes correlated with cognitive decline, suggesting that EEG-based brain network analysis may be useful for early diagnosis and monitoring of dementia progression.

Task-related functional connectivity analyses have provided additional insights into cognitive decline. During working memory tasks, MCI patients exhibit altered connectivity patterns, including decreased fronto-temporal connectivity and increased fronto-occipital and parieto-occipital connectivity in theta and alpha bands (Jiang et al., 2024). Furthermore, decreased alpha band connectivity and lack of beta band modulation with increasing memory load were observed, resulting in a more centralized network structure (Fodor et al., 2021). These changes may reflect compensatory mechanisms in response to neurodegeneration in the hippocampus and surrounding regions. Table 2 summarizes EEG studies of microstate analysis and functional connectivity.

In healthy older adults, high cognitive load tasks are also associated with decreased alpha band connectivity and increased theta band phase synchronization and connectivity (Hou et al., 2018). These findings suggest that graph theory-based functional connectivity analysis during cognitively demanding tasks may reveal characteristic changes in brain functional networks specific to SCD, SMC, and potentially CF.

### 4.3 Novel EEG methods using machine learning and deep learning algorithms

Integrating artificial intelligence with EEG analysis has emerged as a powerful approach for predicting cognitive decline progression. Machine learning algorithms applied to EEG data have high accuracy for classifying AD patients and predicting progression from MCI to AD. For instance, studies using support vector machines and gradient-boosted trees have achieved impressive classification accuracies, reaching 95% for AD detection (Rossini et al., 2022) and 83% for MCI progression prediction in healthy older adults (Mazzeo et al., 2023a). Al-Hagery et al. (2020) improved the accuracy of AD diagnosis to 96.66% using the random forest algorithm as an ensemble method, representing a significant improvement over the single decision tree algorithm (73.33%). These results demonstrate the potential of machine learning techniques, particularly ensemble methods, in enhancing early diagnosis and prediction of dementia progression. The high accuracy achieved by these models suggests their potential clinical application, potentially enabling earlier interventions and more personalized treatment strategies for patients at risk of cognitive decline.

Multimodal approaches combining EEG with other biomarkers may enhance prediction accuracy. Maestú et al. (2019) demonstrated that integrating EEG data with other biomarkers (e.g., genotypes, cognitive tests, or brain imaging) may provide more accurate AD predictions. Kim et al. (2021) developed a model integrating EEG and apolipoprotein E genotypes to predict amyloid positron emission tomography positivity in SCD and MCI, with high accuracy in both groups (see Table 2). These advancements extend early intervention potential to preclinical stages. Mazzeo et al. (2023b) reported a protocol for a prospective cohort study of SCD patients, aiming to develop a model for predicting AD progression using machine learning by integrating multifaceted data including neuropsychological assessments, genetic analysis, EEG, and ERPs.

However, challenges remain in implementing these approaches for large-scale screening, including cost, generalizability, and invasiveness (Rossini et al., 2022). Many studies face limitations, including small sample sizes, short follow-up periods, and difficulties controlling diverse data in multimodal approaches. The variability and reproducibility of machine learning findings across facilities are also concerns. However, in SCD and SMC contexts, machine learning and deep learning models based on large-scale databases are becoming increasingly crucial for distinguishing between actual cognitive impairment and personal cognitive complaints.

## 5 Discussion

Herein, we reviewed the clinical implications of EEG approaches for the early screening of dementia risk in cognitively frail individuals.

Resting-state qEEG is a promising biomarker for SCD, SMC, and possibly CF. When adjusted for cognitive reserve factors, EEG slowing may detect frequency pattern changes and correlate with cognitive decline in high-risk individuals. Combining qEEG with AD pathology markers could enhance its predictive potential for AD progression (Spinelli et al., 2022).

Microstate analysis, functional connectivity analyses, and graph theory approaches may serve as early neural markers of dementia, revealing brain network alterations. These methods, especially when combined with cognitive tasks, can identify subtle functional changes before overt impairments manifest. Recent machine-learning approaches have shown promise in classifying amyloid status in SCD and MCI using EEG features (Kim et al., 2021).

ERP components, particularly P300 and MMN, may detect cognitive frailty in older adults when paired with cognitive tasks. However, their effectiveness is limited in pre-MCI states caused by subtle, multidomain cognitive decline. ERPs are more useful in detecting MCI and AD. As reviewed above, numerous studies have identified common EEG/ERP features in MCI and AD. Combining these with neuropsychological tests and AD biomarkers can improve diagnostic accuracy.

With the increase in young-onset dementia (YOD), EEG has shown potential for YOD diagnosis, particularly in early-onset AD and frontotemporal dementia. Studies highlight distinct EEG patterns, such as increased theta and delta activity in YOD, making EEG a valuable, cost-effective tool for early detection and differentiation (Lin et al., 2021; Brown et al., 2023).

However, clinical application of EEG faces methodological challenges. Evidence for EEG alone to predict dementia progression is insufficient compared with established AD biomarkers (Gouw et al., 2017; Jiao et al., 2023). The absence of standardized guidelines for dementia-specific EEG limits the comparability and generalizability of results (Monllor et al., 2021). Gender differences in dementia risk remain underexplored in EEG research on pre-dementia symptoms despite higher risk in women (Hayden et al., 2006; Chêne et al., 2015). Both EEG and fMRI alone show limited efficacy in distinguishing healthy older adults from MCI (Farina et al., 2020), suggesting the need for multimodal integration (Li et al., 2024).

To overcome these limitations, we propose multi-center collaborative research, such as the “Dementia ConnEEGtome” project (Prado et al., 2022). This approach, with 5-year follow-ups incorporating conventional diagnostic approaches, including AD pathology, could advance standardization, address methodological issues, and improve EEG’s reliability as an early AD biomarker.
